# Targeting focal adhesion kinase in cancer cells and the tumor microenvironment

**DOI:** 10.1038/s12276-020-0447-4

**Published:** 2020-06-09

**Authors:** James M. Murphy, Yelitza A. R. Rodriguez, Kyuho Jeong, Eun-Young Erin Ahn, Ssang-Taek Steve Lim

**Affiliations:** 1 Department of Biochemistry and Molecular Biology, University of South Alabama, College of Medicine, Mobile, AL 36688 USA; 2 Department of Pathology, O’Neal Comprehensive Cancer Center, University of Alabama at Birmingham, Birmingham, AL 35294 USA

**Keywords:** Cancer, Tumour angiogenesis, Tumour immunology, Cancer microenvironment

## Abstract

Focal adhesion kinase (FAK) is an integrin-associated protein tyrosine kinase that is frequently overexpressed in advanced human cancers. Recent studies have demonstrated that aside from FAK’s catalytic activity in cancer cells, its cellular localization is also critical for regulating the transcription of chemokines that promote a favorable tumor microenvironment (TME) by suppressing destructive host immunity. In addition to the protumor roles of FAK in cancer cells, FAK activity within cells of the TME may also support tumor growth and metastasis through various mechanisms, including increased angiogenesis and vascular permeability and effects related to fibrosis in the stroma. Small molecule FAK inhibitors have demonstrated efficacy in alleviating tumor growth and metastasis, and some are currently in clinical development phases. However, several preclinical trials have shown increased benefits from dual therapies using FAK inhibitors in combination with other chemotherapies or with immune cell activators. This review will discuss the role of nuclear FAK as a driver for tumor cell survival as well as potential therapeutic strategies to target FAK in both tumors and the TME.

## Cancer treatment: Targeting more than just the tumor

Targeting a signal protein in the tumor microenvironment (TME), the tissues surrounding tumors, as well as in the tumor itself may lead to more effective cancer treatments. High levels of the signal protein FAK are associated with many tumors. A pro-tumor function of FAK has recently also been reported from the TME, which can strongly influence treatment outcome. Ssang-Taek Steve Lim at the University of South Alabama, Mobile, USA, and co-workers have reviewed the functions of FAK in tumors and the TME. They report that FAK increases development and permeability of blood vessels in the TME, feeding the tumor and permitting cancer cells to travel to other locations. FAK also protects tumors from the immune response and from chemotherapy. These results point the way to treatments potentially effective against many types of cancer.

## Introduction

Focal adhesion kinase (FAK) is a nonreceptor protein tyrosine kinase that is primarily regulated by integrin signaling. Additionally, various transmembrane receptors, including G-protein-coupled, cytokine and growth factor receptors, can coordinate to transmit extracellular signals through FAK^1–3^. FAK controls fundamental cellular processes—cell adhesion, migration, proliferation, and survival^4^, and promotes important malignant features in cancer progression—cancer stemness, epithelial to mesenchymal transition (EMT), tumor angiogenesis, chemotherapeutic resistance, and fibrosis in the stroma^5,6^.

FAK expression is frequently upregulated in different types of cancer, and most studies have focused on either reducing FAK expression or activity to inhibit growth and metastatic capacities of tumors. However, more recent reports suggest that FAK may also contribute to cancer progression by regulating multiple cells or factors within the tumor microenvironment (TME). The TME is the immediate niche surrounding tumors and is composed of blood and lymphatic vessels, immune cells (T and B cells, natural killer cells, and macrophages), stromal cells (fibroblasts, mesenchymal cells, pericytes, and adipocytes), secreted factors and the extracellular matrix (ECM)^7,8^. The tumor and the TME exhibit a remarkable amount of crosstalk that influences cancer progression, metastasis, survival, and the tumor immune landscape^9–11^. While FAK has been mostly investigated in tumors, more recent studies have begun to reveal the role of FAK in the interplay between the tumor and the TME. This review will focus on the roles of FAK signaling in both tumors and the TME, including some recent findings on the role of nuclear FAK in cancer.

## Structure and function of FAK

**Fig. 1:**
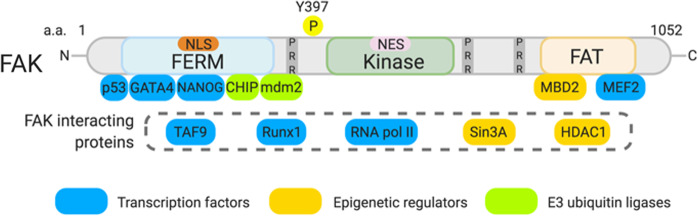
Molecular structure of FAK. FAK comprises three main domains: the FERM (4.1, ezrin, radixin, moesin), central kinase and FAT (focal adhesion targeting) domains. FAK contains both a nuclear localization sequence (NLS) and a nuclear export sequence (NES), which are in the FERM and the kinase domains, respectively. FAK-interacting proteins, including transcription factors, epigenetic regulators, and E3 ligases, are shown. While TAF9, Runx1, RNA pol II, Sin3A, and HDAC1 also interact with FAK, the interacting FAK domain for each remains uncharacterized. Y397: FAK autophosphorylation site. a.a.: amino acids. PRR: proline-rich region. N: N-terminus. C: C-terminus.

FAK is a ubiquitously expressed protein, but its expression in hematopoietic cell lineages is limited. FAK structure can be divided into three main domains: the N-terminal band 4.1, ezrin, radixin, moesin homology (FERM), central kinase, and C-terminal focal adhesion targeting (FAT) domains (Fig. 1). Upon integrin or growth factor receptor signaling, FAK is activated, and FAK autophosphorylation at tyrosine (Y) 397 is increased. Since FAK is a key mediator of integrin signaling through its association with focal adhesion proteins, such as talin and paxillin, it has largely been thought that FAK localization might be limited to the cytosol and plasma membrane. However, this idea was later challenged by the identification of a functional nuclear localization sequence (NLS) within the FAK FERM domain and a nuclear export sequence (NES) in the central kinase domain (Fig. 1)^12,13^. The NLS and NES enable FAK to constantly shuttle between the cytosol and nucleus, which has since expanded the scope of FAK signaling to the regulation of nuclear proteins and gene expression. Although the role of nuclear FAK is not fully understood, several studies have shown that nuclear FAK may act as a key player in regulating gene expression by interacting with numerous transcription factors (NANOG, TAF9, MEF2, RUNX1, and RNA polymerase II), E3 ligases (mdm2 and CHIP) and epigenetic regulators (HDAC1, MBD2, and Sin3a) (Fig. 1)^13–18^. Earlier nuclear FAK studies demonstrated that the FERM domain acts as a scaffold to promote ubiquitination and proteasomal degradation of nuclear factors (e.g., p53 and GATA4) by forming a complex with E3 ligases (e.g., mdm2 and CHIP) (Fig. 1)^13,14,19^. In cell culture conditions, FAK primarily localizes to the cytosol and focal contacts; however, we found that FAK is predominantly localized to the nucleus in smooth muscle cells of healthy arteries^14^, suggesting that FAK localization may differ in vivo and in vitro.

## The roles of nuclear FAK in cancer

FAK functions can be broadly separated into two categories: cytosolic and nuclear. Cytosolic FAK functions include signaling cascades of transmembrane receptors, which enhance focal adhesion turnover, cell adhesion, cell migration, and gene expression in response to extracellular signals. FAK’s cytosolic signaling functions in cancer cells are heavily dependent on increased FAK activity. In advanced human cancers, FAK overexpression by FAK gene amplification or mRNA upregulation is often associated with increased FAK activation, resulting in poor clinical prognosis^5,20^.

On the other hand, the discovery of nuclear FAK regulation of gene expression has added another layer of complexity to FAK signaling. Identifying nuclear FAK regulation of the tumor suppressor p53 stability was the first to demonstrate the importance of nuclear FAK function in cell survival and gene expression^13^. Further characterization of FAK nuclear localization demonstrated a series of favorable conditions that promote FAK nuclear localization, including conditions related to loss of cell attachment and apoptosis^13^. These conditions are commonly associated with loss of FAK catalytic activity, and indeed, FAK catalytic inhibition by small molecule FAK inhibitors or genetic FAK kinase-dead (KD) mutations significantly promote FAK nuclear localization^19^. The FAK KD knock-in model further supports a kinase-independent scaffold function of nuclear FAK in mice and mouse embryonic fibroblasts (MEFs) to regulate proinflammatory signaling. In MEFs, nuclear FAK enhances degradation of the GATA4 transcription factor and blocks the expression of vascular cell adhesion molecule-1 (VCAM-1) upon tumor necrosis factor-α (TNF-α) stimulation^19^. This study suggested that nuclear FAK may function as an anti-inflammatory signal.

New molecular mechanisms of nuclear FAK function in tumor progression have been further investigated using skin squamous carcinoma cells (SCCs), triple-negative breast cancer cells (TNBCs), and melanoma cells. Interestingly, FAK-knockout SCCs re-expressing WT FAK exhibited abundant nuclear FAK in contrast to the lack of nuclear FAK in normal keratinocytes^21^. In SCCs, nuclear FAK promotes the expression of several chemokines, including CCL5 and TGFβ2, which promote an immunosuppressive TME^21^. Interestingly, SCCs expressing either FAK KD (inactive but nuclear localized) or FAK NLS mutants (active but cytosol restricted) failed to promote CCL5 expression, suggesting that nuclear FAK may exhibit some catalytic activity required for CCL5 transcription. CCL5 gene expression potentially occurs through FAK interaction with TAF9, which is part of the transcription factor II D complex that makes up the RNA polymerase II preinitiation complex. However, the exact mechanism by which nuclear FAK regulates TAF9 to promote gene expression is not known. In another study with SCCs, nuclear FAK was shown to downregulate the expression of insulin-like growth factor binding protein 3 (IGFBP3), a tumor suppressor, by promoting the interaction between the transcription factor RUNX1 and the transcriptional suppressor Sin3a^17^. Interestingly, suppression of IGFBP3 transcription was independent of FAK catalytic activity and was solely due to a kinase-independent role of nuclear FAK^17^. This finding is contradictory to previous observations in which active nuclear FAK promoted SCC immunosuppression^21^. Although it has not been clearly demonstrated that nuclear FAK is active in SCCs, it will be intriguing to identify any target proteins phosphorylated by nuclear FAK.

Several studies have indicated that FAK and NANOG (a transcription factor critical for stem cell pluripotency) regulate each other to promote an aggressive tumor phenotype. In colon cancer cells, NANOG was shown to bind the FAK promoter and increase FAK expression^22^. NANOG was then found to associate with and be phosphorylated by FAK, and increased FAK-NANOG complexes promoted cancer cell pluripotency and invasive capacity. The FAK FERM domain is responsible for NANOG binding, and mutation of Y35F and Y174F (tyrosine to phenylalanine) of NANOG disrupted the FAK-NANOG association and reduced cancer cell invasiveness^22^. FAK and NANOG were shown to colocalize to the nucleus, but the Y35F and Y174F NANOG mutants showed decreased nuclear localization, suggesting that FAK phosphorylation and interaction may regulate NANOG nuclear translocation and transcriptional activity in colon cancer cells. FAK and NANOG were further shown to interact within the cancer stem cell (CSC) population of TNBC cells through their interaction with connexin 26 (Cx26), a cell-cell adhesion molecule^15^. The interaction between FAK and NANOG in the nucleus and cytoplasm was unique to TNBC CSCs and did not occur in non-CSCs. In normal epithelial or luminal breast cancer cells, FAK and NANOG did not bind each other but did associate with Cx26. In TNBC CSCs, Cx26 seems to increase both NANOG protein stability and FAK activity. While FAK is known to phosphorylate NANOG in colon cancer cells, FAK activity was not required for the formation of the Cx26/FAK/NANOG complex in TNBC CSCs^15^. Using human breast cancer datasets, this study further found that TNBC patients with higher levels of Cx26/FAK/NANOG had decreased relapse-free survival compared to those with lower expression. While these studies suggest that nuclear FAK within cancer stem cells helps promote an aggressive phenotype, it is still not clear why or how nuclear FAK is increased within some types of cancer.

Homozygous Y397F (tyrosine to phenylalanine)-mutated FAK knock-in mice were embryonic lethal at E9.5 and showed a similar phenotype to fibronectin-deficient embryos^23^. Mass spectrometry analysis of proteins that bind the Y397-containing peptide in the FERM-kinase linker (Fig. 1) found that only myosin-1E (Myo1E), an actin-based molecular motor, bound both the phosphorylated and nonphosphorylated Y397-containing peptides. In melanoma cells, Myo1E binding to FAK increases FAK activity and promotes FAK nuclear localization. Nuclear FAK promoted the expression of several ECM proteins, such as osteopontin and fibronectin, that drive melanoma proliferation^23^. The researchers also demonstrated that pharmacological FAK inhibition reduced pY397 FAK and reduced nuclear FAK, showing that FAK activity in human melanoma may be important for FAK nuclear localization. However, the study did not reveal the mechanism by which FAK activity contributes to the nuclear localization of FAK or ECM gene expression.

More recent studies have focused on investigating the subcellular localization of both total and active FAK within various cancer specimens. Using large numbers of human tumor samples, the studies showed nuclear staining of active phosphorylated Y397 (pY397) FAK within several types of cancers, including lung, colorectal, and breast cancer^24–26^. Interestingly, the colorectal and breast cancer studies showed that elevated nuclear pY397 FAK was associated with a poor prognosis and decreased patient survival^25,26^. Patient survival in both small-cell lung and non-small-cell lung cancers did not correlate with increased levels of nuclear pY397 FAK^24^, suggesting that nuclear pY397 FAK may play different roles in the progression and aggressiveness of different tumor types. Human ovarian cancers also showed increased pY397 FAK in tumor cells compared to stromal cells, and pY397 FAK was widely distributed in both the cytosol and nucleus;^27^ however, this study did not investigate the role of nuclear FAK in ovarian cancer or any associations with patient survival. Although these immunohistochemical analyses of human cancers have provided new insights into the prevalence and potential importance of active nuclear FAK within tumor cells, more comprehensive analyses of nuclear FAK localization and activity in various types of tumors are needed to better understand FAK’s role in the nucleus of cancer cells.

Overall, these studies suggest that both kinase-dependent and kinase-independent roles of nuclear FAK may be required for the regulation of cancer cell survival and aggressiveness. More studies are still needed to elucidate how nuclear FAK with or without catalytic activity drives tumor progression in various cancers. Interestingly, we have observed that unlike FAK in cancer cells, FAK in vascular smooth muscle cells (VSMCs) in vivo is predominantly in the nucleus and does not appear to be active^14^, suggesting that cancer cells may use unknown mechanisms to promote nuclear FAK activity.

The potential differences in the status of active nuclear FAK between VSMCs and cancer cells could come from the differential expression of total FAK and active pY397 FAK levels. As FAK expression and activity are often increased in a number of cancers, it is possible that the abundance of active FAK increases the chance of there being active pY397 FAK within the nucleus.

## FAK-mediated interactions between tumors and the TME

**Fig. 2:**
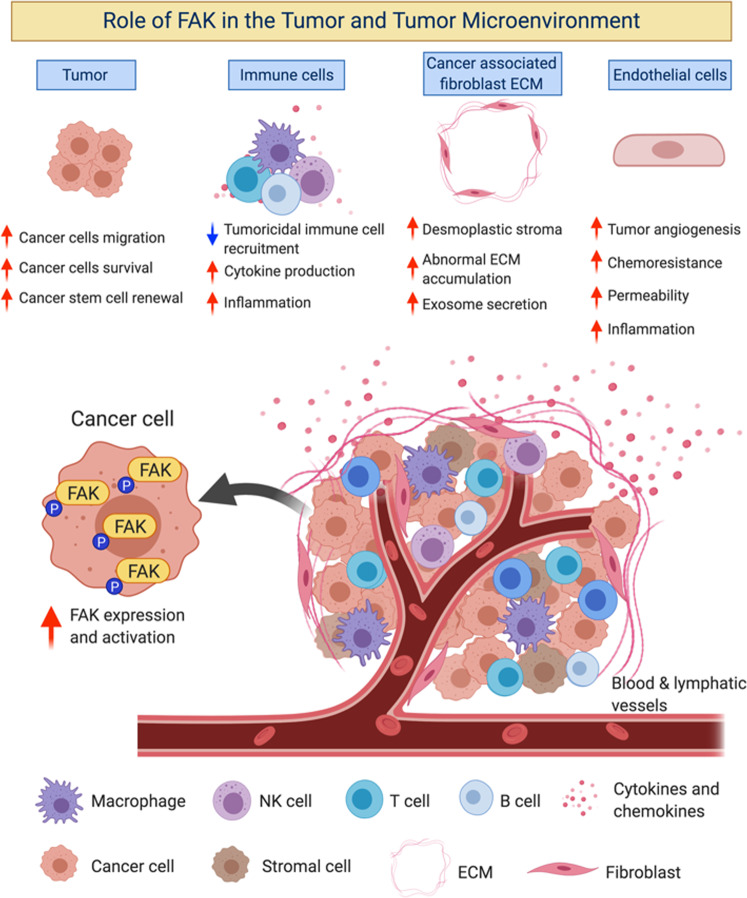
Role of FAK in the tumor and the tumor microenvironment (TME). The TME comprises a heterogeneous population of cells and acellular components. The orchestration of signaling pathways and communication between cell populations within the TME significantly dictates the fate of tumor growth and development. FAK has been shown to play an important role in the regulation of tumor and TME functions to provide a favorable and protumorigenic niche.

TME comprises a heterogeneous population of cells (endothelial cells, immune cells, stromal cells, and fibroblasts) and acellular components (ECM, cytokines/chemokines, growth factors, and hormones) that surround tumors (Fig. 2). It is well known that the TME plays a crucial role in tumor initiation, progression, and metastasis^7,8^. The TME is maintained by a complex interplay between cells and signaling cascades influenced by cancer cells and between TME components. Recent studies have demonstrated the role of FAK in promoting TME remodeling, including roles in angiogenesis, immune cell recruitment, and ECM production, which exacerbate tumor progression (Fig. 2). Targeting FAK in both the tumor and TME could prove beneficial in reducing tumor-TME interactions and reducing tumor progression.

### Immune cells

Immunotherapy using the host immune system to target cancer cells has become increasingly popular over the last several years. Tumors secrete various cytokines, chemokines, and extracellular matrix proteins that can lead to an immunosuppressive environment, thus promoting tumor cell survival^28^. A recent study showed that SCC exhibits an abundant level of nuclear FAK. Loss of FAK expression in SCCs promoted tumor regression, and re-expression of WT FAK, but not KD FAK, promoted tumor progression^21^. The study found that nuclear FAK increased the expression of CCL1, CCL5, CCL7, CXCL10, and TGFβ2, chemokines and cytokines responsible for recruitment and expansion of regulatory T cells (Tregs)^21^. The recruited Tregs promoted tumor survival by exhausting the CD8^+^ cytotoxic T cell population responsible for tumor cell clearance^29^. In mice bearing SCC tumors, pharmacological FAK inhibition led to a decrease in Treg recruitment and an increase in cytotoxic CD8^+^ T cells^21^, demonstrating that pharmacological FAK inhibitors can promote active immune surveillance in the TME to prevent tumor progression. In a pancreatic ductal adenocarcinoma (PDAC) mouse model, FAK activity and expression also promoted an immunosuppressive TME by increasing the recruitment of Tregs^30^, suggesting that FAK in cancer cells plays a key role in regulating the tumor immune landscape. A follow-up study showed that nuclear FAK enhanced the gene expression of interleukin-33 (IL-33), an IL-1 family cytokine that can be secreted or localized to the nucleus. Nuclear FAK interacts with IL-33 to promote transcription of the soluble ST2 receptor (sST2), for which IL-33 is a ligand^16^. Increased sST2 then blocked recruitment and activation of CD8^+^ cytotoxic T cells by acting as a sink for extracellular IL-33^16^. The FAK-IL-33 complex was also shown to interact with the chromatin modifiers, WDR82 and BRD4, suggesting that nuclear FAK may help promote an open chromatin structure to increase transcription of chemokine genes in SCCs. These studies revealed that both nuclear FAK and its activity may be important for the transcription of different chemokines, which may potentially occur through FAK’s association with WDR82, BRD4 and the transcription factor TAF9. However, the molecular mechanism by which FAK activity in the nucleus contributes to chemokine transcription is unclear.

T cells require costimulatory receptor activation to become fully active and promote tumor cell clearance. A recent study demonstrated that cancer cells expressing CD80, a T cell costimulatory ligand, were more sensitive to pharmacological FAK inhibition and clearance by CD8^+^ cytotoxic T cells than cancer cells without CD80 expression^31^. Cells expressing CD80 can induce cytotoxic T cell activation through association with CD28, a T cell costimulatory receptor, which promotes tumor clearance^32^. Although the mechanistic signaling pathways remain elusive, FAK inhibition induced tumor regression of CD80-expressing tumors by increasing the CD28^+^ T cell population within the TME. FAK inhibition was also able to promote clearance of CD80-deficient tumors when combined with agonistic antibodies against T cell costimulatory receptors such as OX-40 and 4-1BB^31^. These studies highlight the potential benefits from the combined use of FAK inhibitors and immunotherapy in terms of altering the T cell population of the TME to promote tumor regression.

Immune cells can play a key role in promoting either tumor survival or clearance^33^. The recruitment of various types of immune cells, such as Tregs, CD4^+^ T cells, CD8^+^ T cells, tumor-associated macrophages, and natural killer cells, plays a distinct role in promoting either tumor cell survival or clearance. A recent study illustrated the importance of FAK expression within myeloid cells of the TME. In an MMTV-polyoma middle T murine model of breast cancer, FAK was knocked out in mononuclear phagocytic cells using LysM-Cre^34^. Interestingly, the group with loss of FAK expression in myeloid cell lineages had delayed formation of adenomas and carcinomas compared to the WT control group. However, in established tumors, myeloid cells with FAK knockout showed more accelerated tumor growth than the WT FAK cells^34^. This increase in tumor size was associated with decreased natural killer cells within the tumor, suggesting that FAK expression in myeloid cells is important for natural killer cell recruitment and/or survival within the TME^34^. While this study used a FAK knockout model, further studies are needed to investigate the impact of FAK catalytic activity and nuclear localization in myeloid cells on the recruitment of other immune cells to the TME.

### Endothelial cells (ECs)

FAK expression is upregulated in vascular cells surrounding solid tumors^35,36^. Numerous studies have investigated the role of FAK within endothelial cells (ECs) in tumor survival, angiogenesis, and metastasis. EC-specific deletion of FAK, just prior to tumor implantation, demonstrated that FAK expression is required for vascular endothelial growth factor (VEGF)-induced angiogenesis and subsequent tumor growth^37^. The importance of FAK catalytic activity in ECs during tumor formation was further investigated by using the autophosphorylation mutant FAK Y397F (tyrosine to phenylalanine), which lacks catalytic activity. EC-specific expression of the FAK Y397F mutant reduced the initiation of tumor angiogenesis, suggesting that FAK activity or activation in ECs is important for angiogenesis to support tumor growth^38^. ECs isolated from FAK Y397F mice showed decreased VEGF receptor 2 (VEGFR2) expression and β1 integrin activation^38^. Interestingly, another study revealed that FAK nuclear localization and kinase activity were critical for VEGFR2 transcription and that FAK bound the VEGFR2 promoter following VEGF treatment in mouse ECs^39^. These studies shed light on the importance of investigating the role of nuclear FAK and potential nuclear FAK activity in ECs during tumor angiogenesis.

Increased vascular permeability promotes tumor metastasis by making it easier for cancer cells to enter and exit the bloodstream. FAK expression has been demonstrated to promote metastasis through increased vascular permeability and disorganized vessel structure^40^. Loss of FAK expression within ECs increased both cell-cell junctions and astrocyte-endothelium interactions, which led to decreased permeability within glioma^40^. More importantly, it has been shown that FAK activity in ECs promotes tumor cell metastasis by promoting vascular permeability through VEGF signaling^41^. Mechanistically, VEGF promotes FAK activation, rapid FAK localization to cell-cell junctions, binding of the FAK FERM domain to vascular endothelial cadherin (VE-cadherin), and direct phosphorylation of β-catenin at Y142, facilitating EC junction breakdown by dissociating the VE-cadherin-β-catenin complex. By using an EC-specific FAK KD model, studies have found that FAK catalytic activity within ECs plays an important in disrupting the EC barrier not only by phosphorylating β-catenin at Y142 but also by phosphorylating Y658 on VE-cadherin^42^. While these studies focused on the loss of FAK catalytic activity within the cytoplasm, the role of nuclear FAK in the regulation of vascular permeability needs further investigation as FAK KD mutant can localize to the nucleus^14^.

Metastasis is a complex process that involves tumor cell intravasation, subsequent attachment to the distal endothelium, and finally extravasation into secondary tissue. In addition to blood vessels, tumors also metastasize through lymphatic vessels. Thus, proximal lymph nodes are often identified as the primary sites of metastasis. Our recent study showed that lymph node ECs express VCAM-1, which is important for melanoma-lymphatic EC interactions^43^. FAK inhibition reduced VCAM-1 expression both in lymph nodes and human dermal lymphatic ECs, which was associated with reduced lymph node metastasis and melanoma-EC attachment^43^. These studies demonstrate that FAK plays important roles in both vascular and lymphatic ECs in the promotion of both tumor growth and subsequent metastasis.

Tumors can develop resistance to chemotherapies, which can make them more aggressive and lead to decreased patient survival. The TME can promote chemoresistance by providing a favorable environment for cancer cells. FAK expression in ECs has been shown to protect tumor cells from DNA-damaging therapies such as doxorubicin and irradiation^44^. However, loss of FAK increased cancer cell sensitivity to DNA-damaging therapies, potentially through the loss of EC-derived cytokines and chemokines that induce survival signaling within the tumor cells. Mechanistically, it seems that EC FAK is required for DNA damage-induced NF-κB activation and cytokine production. While this study suggested that FAK expression in ECs is important for promoting chemoresistance, it is still unknown whether this occurs through FAK catalytic activity or through its kinase-independent scaffolding functions.

### Cancer-associated fibroblasts (CAFs) and ECM

Cancer-associated fibroblasts (CAFs) are the primary stromal cells found within the TME. CAFs help facilitate pleiotropic events, including tumor cell initiation, survival, proliferation, and metastasis, through the production and secretion of various cytokines, growth factors, hormones, and ECM proteins^45^. Increased expression of the ECM protein lumican by gastric CAFs was shown to activate FAK via β1 integrin to promote the proliferation, migration and invasion potential of gastric cancer cells^46^. PDAC cells have been shown to activate CAFs and promote the expression of type I collagen^47^. Increased type I collagen expression promoted cancer stemness of PDAC cells through β1 integrin-FAK signaling^48^. Pharmacological inhibition or shRNA knockdown of FAK in PDAC cells reduced CAF recruitment and fibrosis in the TME^30^. The importance of FAK in PDAC cell stemness was further supported by overexpression of either active WT or inactive Y397F-mutated FAK in PDAC cells. Overexpression of WT FAK, but not FAK Y397F, led to increased tumor incidence even when as few as 500 cells were injected into severe combined immunodeficient (SCID) mice, suggesting that FAK activity is important to drive cancer cell stemness and recruitment of CAFs^48^. FAK inhibition reduces CAFs within PDAC tumors and increases PDAC resistance to FAK inhibitors^49^. This chemoresistance was found to be due to decreased secretion of TGF-β by CAFs, which led to increased activation of STAT3 signaling^49^. The importance of FAK in CAFs was further studied through the use of a fibroblast-specific FAK knockout mouse model in which the loss of FAK in CAFs led to decreased breast cancer metastasis^50^. This study found that exosomes isolated from FAK-null CAFs contained elevated levels of the tumor-suppressing microRNAs miR-16 and miR-148a^50^, suggesting that FAK expression in CAFs may play a role in exosome-mediated regulation of cancer cell proliferation. Further studies are needed to evaluate the importance of FAK activity and/or localization on CAFs in tumor progression, survival and metastasis.

## FAK inhibitors in dual therapy

As FAK appears to play such an important role in fundamental cellular processes during tumor progression, FAK has become an attractive target for cancer therapies. The earliest efforts used to block FAK signaling include inactivation via the expression of antisense oligos or via overexpression of the FAK C-terminal domain^51,52^. More recent efforts have focused on the generation of small molecule inhibitors (and peptides to a minor extent) that block FAK activity using diverse mechanisms. Despite various FAK inhibitors showing acceptable phase I safety profiles^53–56^, multiple reports have suggested that FAK inhibitors can induce cell cycle arrest or apoptosis of cancer cells. Some FAK inhibitors have moved into phase II clinical trials as part of combinatorial treatments with either other pharmacological inhibitors or blocking antibodies in multiple solid tumors, including pancreatic cancer and non-small-cell lung carcinoma (clinical trial identifiers: NCT02758587 and NCT02546531, respectively).

While chemotherapies have shown benefits in reducing tumor burden, some tumors are remarkably resistant or can develop chemoresistance. Adding pharmacological FAK inhibitors to current pharmacological therapies results in a profound effect on tumor remission (Table 1). BRAF mutations, with BRAF V600E being the most prominent, are highly prevalent in both melanoma and colorectal cancers^57^. BRAF inhibitors led to hyperactivation of FAK and subsequent upregulation of the Wnt/β-catenin signaling pathway in BRAF-mutant colorectal cancer cells^57^. While it is currently unknown how these BRAF inhibitors lead to increased FAK activation in these BRAF-mutant cells, the use of a FAK inhibitor in combination with BRAF inhibitors decreased tumor growth more than the use of either inhibitor alone^57^. In pancreatic cancer, WT1-associated protein (WTAP) promotes chemoresistance to gemcitabine by binding to FAK mRNA and increasing FAK expression and activity^58^. While cotreatment with a FAK inhibitor and gemcitabine showed increased efficacy in WTAP-expressing pancreatic cancer cells compared with the use of either therapy alone^58^, this study did not test if the same results occur in vivo. Paclitaxel-resistant ovarian cancer develops resistance through YB-1 (a transcription factor)-mediated expression of the cancer stem cell marker CD44^59^. FAK inhibition was able to overcome paclitaxel resistance by reducing CD44 expression through decreased YB-1 activation and nuclear translocation. Another study found that ovarian cancer with intrinsic or acquired resistance to platinum-based therapies could also be targeted through cotreatment strategies with a FAK inhibitor^27^. Overall, these studies demonstrate that several cancers are able to develop resistance to various therapies and that treating these cancers with mutliple anti-cancer agents including FAK inhibitors could prove useful.

**Table 1:** Preclinical trials using FAK inhibitors in dual therapy.

Cancer	Inhibitor	Target	References
Ovarian	VS-4718	FAK inhibitor	^27^
	Paclitaxel and cisplatin	Cell cycle inhibitor	
Ovarian	VS-6063	FAK inhibitor	^59^
	Paclitaxel	Cell cycle inhibitor	
Lung	Tanespimycin	HSP90 inhibitor	^64^
	PF-573228	FAK inhibitor	
Mesothelioma	VS-4718	FAK inhibitor	^65^
	Pemetrexed	Nucleotide synthesis inhibitor	
	Cisplatin	DNA-damaging agent	
Skin	Vorinostat or Panobinostat	HDAC inhibitor	^66^
	VS-4718	FAK inhibitor	
Colorectal	PF-562271	FAK inhibitor	^57^
	Vemurafenib	BRAF inhibitor	
	Trametinib	MEK1/2 inhibitor	
Pancreatic	GSK2256098	FAK inhibitor	^58^
	Gemcitabine	Blocks DNA replication	
Pancreatic	VS-4718	FAK inhibitor	^30^
	Gemcitabine	Blocks DNA replication	
Pancreatic	VS-4718	FAK inhibitor	^49^
	Stattic	STAT3 inhibitor	

The use of immunomodulating agents in combination with FAK inhibitors has also gained traction in preclinical settings (Table 2). Several cell surface proteins, such as programmed cell death protein 1 (PD1), programmed death-ligand 1 (PD-L1), and cytotoxic T lymphocyte-associated protein 4 (CTLA4), are known to promote cancer cell survival through evasion of the immune system^60^. A recent study showed that treatment with neutralizing antibodies against PD1 and CTLA4 in combination with a FAK inhibitor significantly reduced pancreatic tumor size and increased survival rates in pancreatic cancer mouse models^30^. Another study demonstrated that PD-L1-neutralizing antibodies in combination with a FAK inhibitor also promoted cytotoxic T cell activation and decreased the number of TNBCs^61^. While cancer cells expressing CD80, the receptor for the T cell inhibitory protein CTLA4, showed remarkable sensitivity to FAK inhibitors, cancer cells with low levels of CD80 were resistant to FAK inhibition^31^. However, combination therapy using a FAK inhibitor and agonistic antibodies against OX-40 or 4-IBB, receptors important for T cell activation, was able to overcome CD80-negative cancer cell insensitivity to FAK inhibition. Taken together, these studies suggest that FAK plays a key role in protecting cancer cells from clearance by the immune system and implicate FAK as an attractive target in immunomodulating dual therapies.

**Table 2:** Preclinical trials using FAK inhibitors in combination with immunomodulating antibodies.

Cancer	Inhibitor	Target	References
Breast	PF-573228	FAK inhibitor	^61^
	Atezolizumab	Anti-PD-L1 antagonistic antibody	
Pancreatic	VS-4718	FAK inhibitor	^30^
	Antagonistic antibodies	Anti-PD1 and Anti-CTLA4	
Pancreatic and skin	BI 853520	FAK inhibitor	^31^
	Anti-OX-40 or anti-4-IBB antibodies	CD8^+^ T cell activator	
	Gemcitabine	Blocks DNA replication	

## Conclusion

The dynamic interplay between tumors and the TME gives rise to a very protumorigenic environment, and tumors and the TME can even influence each other in the development of chemoresistance. Herein, we have outlined several promising studies that have evaluated how FAK plays important roles in both tumors and the TME in promoting tumor progression and chemoresistance. While several clinical phase I and II trials are currently underway using FAK inhibitors in combinatorial therapies, more research needs to be done to better understand how FAK regulates both tumors and the TME. Most FAK inhibitors under development also inhibit proline-rich tyrosine kinase 2 (Pyk2), the only other member of the FAK family. While Pyk2 does have some overlapping functions with FAK and has been investigated in some cancers, including multiple myeloma^62,63^, more studies are needed to investigate the role of Pyk2 in both tumors and the TME. Additionally, we have highlighted the growing importance of nuclear FAK in tumor progression and survival and demonstrated that some cancers feature abundant active nuclear FAK, which is not typically observed in other cell types. Despite all the evidence described in this review, the roles of nuclear FAK in tumors and the TME remain elusive. Comprehensive future studies of nuclear FAK function will provide novel knowledge about the importance of the FAK signaling axis in the TME and tumor progression. Clear elucidation of nuclear FAK signaling will identify novel oncogenic and tumorigenic targets of nuclear FAK with the hope of finding novel therapeutic agents. The development of preclinical models to study the role of nuclear FAK and the relationship between tumors and the TME is of immediate need.
